# ESPRES: A web application for interactive analysis of multiple pressures in aquatic ecosystems

**DOI:** 10.1016/j.scitotenv.2020.140792

**Published:** 2020-11-20

**Authors:** Angel Udias, Alberto Pistocchi, Olga Vigiak, Bruna Grizzetti, Faycal Bouraoui, Cesar Alfaro

**Affiliations:** a European Commission – Joint Research Center, via E. Fermi 2749, 21027 Ispra, VA, Italy; b Universidad Rey Juan Carlos, 28933 Mostoles, Madrid, Spain

**Keywords:** Web-based decision support system, Multi-objective optimization, Multiple pressures management, Water management, River Basin planning, Trade-off analysis

## Abstract

**ESPRES** (***E***fficient ***S***trategies for anthropogenic ***P***ressure ***R***eduction in ***E***uropean water***S***heds) is a web-based Decision Support System (DSS) designed to explore management options for achieving environmental targets in European freshwaters. The tool integrates multi-objective optimization (MOO) algorithms for selecting the best management options in a river basin and models assessing the consequent changes in the water quantity (water flow) and quality (nutrient concentration). The MOO engine identifies Pareto front strategies that are trade-offs between environmental objectives for water bodies and the effort required for reducing the pressures. The web interface provides tools to set the effort perceived by different river basin stakeholders considering technical feasibility, political difficulty, and social acceptability of the alternative options. The environmental impact of management options (scenarios) is assessed with models developed at the European scale. ESPRES enables comparison of management solutions and allows quantifying environmental and socio-economic trade-offs inherent to the decision making process.

## Highlights

• ESPRES helps exploring management options for reducing pressures in European basins.
• Stakeholder (web users) tailor water management analysis based on their knowledge.
• ESPRES identifies trade-offs between environmental outcomes and perceived effort.
• Environmental outcomes are based on scientific models, effort on user knowledge.
• Interactive web-tools support transparent decision-making based on science.

## Graphical abstract

**Figure:**
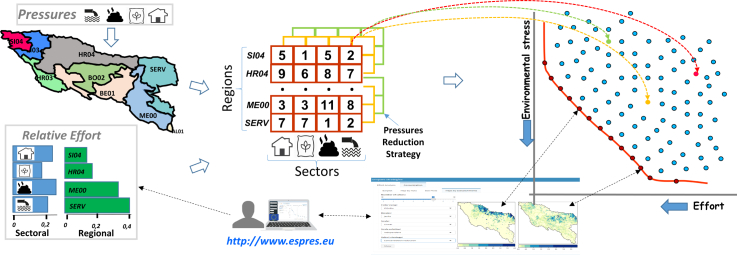

## 1 Introduction

The European Union Water Framework Directive (WFD) 60/2000/EC (European Parliament and Council, 2000) requires Member States to set out river basin management plans to ensure the good ecological status of the water bodies. When the status is less than “good”, the responsible anthropogenic pressures must be identified, and adequate Programmes of Measures (PoM) must be put in place to restore water bodies conditions. In this context, river basin management planning requires an assessment framework making explicit the causal relations between pressures and water bodies status, in order to support the design of effective and adequate programmes of measures, with due consideration of costs and benefits for the basin communities and the environment (Pistocchi et al., 2017).

River basin management cannot be simply treated as a technical question with a univocal answer (Rittel and Webber, 1973). Aquatic ecosystems are threatened by a variety of pressures, such as water abstraction, organic and inorganic pollution, invasive species, pathogens, geomorphological alterations, land use and climate variability and change (Vörösmarty et al., 2010). These pressures often act simultaneously, with synergistic or antagonistic effects (Coors and De Meester, 2008; Ormerod et al., 2010; Kalogianni et al., 2017). Additionally, water fluxes and aquatic ecosystems are extremely heterogeneous in space and time, hence water resources management is intrinsically complex (Stevenson and Sabater, 2010).

Furthermore, human lives and activities rely on limited water resources. Thus, basin management entails an argumentative process (Kunz and Rittel, 1970) whereby the community interrogates itself on the desired conditions of the river basin and potential management actions. The general public is paying increasing attention to environmental matters; groups and individual citizens are becoming actors in planning and decision-making processes (Giupponi and Sgobbi, 2013). Indeed, management strategies must be questioned and discussed openly; aspirational goals must be compared to what can be realistically achieved, and solutions must not only be technically feasible but also socially acceptable. To engage communities and identify solutions that best fulfil expectations and comply with legislative requirements, an open discussion with all stakeholders must be established from the onset of the planning work (Kunz and Rittel, 1970). Stakeholders' engagement enhances the relevance of plans to local needs at farm and catchment scale, and foster better policies and incentive schemes (Hashemi et al., 2016; Voinov et al., 2016).

Decision Support Systems (DSSs) may help water resource managers in several tasks such as: (i) assessing current issues and analyzing cause-effect (pressure-impacts) relationships; (ii) exploring potential strategies, and (iii) engaging stakeholders in participated and transparent decision-making. DSSs increasingly rely on hydro-ecological, social, and economic modelling to identify patterns, explore options, and project potential outcomes of management plans (Zalewski et al., 1997; Matthies et al., 2007; Harou et al., 2009; Sivapalan et al., 2012). On the downside, DSSs are typically difficult to use, thus exploration of scenarios remains restricted to a small number of options developed along informative narratives (Berntsen and Trutnevyte, 2017; Alcamo, 2008; McInerny et al., 2014). In this process, interaction with stakeholders is often restricted to consultations in the initial and final phases of the modelling exercise.

Conversely, environmental models can potentially generate hundreds of scenarios, providing powerful opportunities to explore efficient strategies when the planning exercise seeks to meet many objectives at once. Multi-objective optimization (also known as Pareto optimization) methods in connection with environmental models proved effective in addressing contrasting management goals (Udias et al., 2016). Particularly in water resources management, Multi-Objective Evolutionary Algorithms (MOEA) coupled with biophysical models have shown that tuning actions to the local context and accounting for downstream impacts may generate large savings while ensuring positive environmental outcomes (Maier et al., 2014; Udias et al., 2018). These potentials are often overlooked in the absence of optimization analysis (e.g. Hashemi et al., 2016).

The effectiveness of DSSs in stakeholder engagement depends on (i) the relevant actors and their roles, (ii) the knowledge characteristics to be transferred, as well as (iii) the interface through which knowledge is transferred (Booty et al., 2001; Holmes and Clark, 2008). Interactive web-tools that provide access to scientific methods via user-friendly interfaces have proven effective in supporting transparent decision-making based on science (Sandink et al., 2016). The advantages of interactive web-tools as compared to more traditional scientific outputs are multiple. Whereas traditional scientific outputs show “static” visualizations and explanations of data or model outputs, interactive web-tools offer control over it, and promote active exploration and understanding of this information (McInerny et al., 2014). Interactive web-tools may also be useful for customizing information to different audiences (De Carolis et al., 2016; Guivarch et al., 2017).

In this paper, we present **ESPRES** (***E***fficient ***S***trategies for anthropogenic ***P***ressure ***R***eduction in ***E***uropean water***S***heds), a web-based DSS designed to help stakeholders exploring management options for reducing the impact of anthropogenic pressures in European river basins. ESPRES combines a flexible modelling framework with a user-friendly interface that enables assessing the potential improvement brought by river basin management strategies to achieve environmental goals. The EDSS is designed to allow comparative analysis of management strategies and to identify trade-offs between environmental and socio-economic goals. The paper is organized as follows. Section 2 discusses the multi-objective problem, ESPRES approach, architecture and features. Section 3 introduces the critical interactive steps of the ESPRES analysis flow. Section 4 provides an example of usage of ESPRES demonstrating visualization tools and options. Section 5 concludes by providing recommendations for further enhancements to ESPRES.

## 2 ESPRES: a web-tool for multi-objective river basin planning

Integrated river basin management must meet environmental targets while preserving the economic activities of its communities. In general, we expect the existence of trade-offs between the improvement of the condition of water bodies due to a reduction of pressures, and the effort entailed for achieving it (e.g. Pistocchi et al., 2017).

**Fig. 1:**
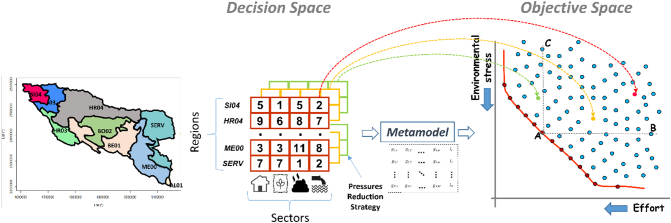
The search for optimal pressure reduction strategies: an example of nutrient reduction in the Sava basin. From left to right: (i) decision variables depends on administrative regions of the basin; (ii) an example of management strategy (table) in the decision space: the columns correspond to water pressure sources (four sectorial nutrient pollution); the rows refer to the administrative units; the values in the table are the rates of reduction of nutrient pollution (0–100%) of the strategy. (iii) Mapping of the strategy in the objective space by evaluating its outcomes in terms of effort and environmental impact. (iv) The two-dimensional objective space (Environmental stress vs Effort), in which Pareto optimal strategies defines the border of what can be achieved: point A belongs to the Pareto front of minimizing it achieves the same environmental outcome of point B but with a lower effort, and it achieves a larger environmental outcome than point C for the same effort.

Optimal trade-off solutions belong to a so-called Pareto front, i.e. the group of solutions that obtain the maximum impact for a given effort. All other solutions (not Pareto-optimal) either achieve less or cost more than the optimal ones. In a two dimension objective space (two axis graph), Pareto front solutions lay along a line (Fig. 1). All solutions in the Pareto front are optimal, however they may achieve more or less environmental outcomes depending on the effort spent to achieve it. Furthermore, it is usually possible to identify an effort level above which the incremental impact becomes less than proportional to the incremental effort. The choices of what effort can be sustained or what environmental result can be sufficient belong to decision makers.

### 2.1 Management strategies to reduce environmental pressures

ESPRES allows assessing environmental outcomes versus difficulties in tackling basin pressures. The efficiency of alternative management strategies to reduce pressures on water is evaluated in terms of the achievements of two contrasting objectives, an environmental one and a socio-economic one (effort).

A management strategy consists of a set of decision variables on the rate of pressure reduction per sector (pressure source) and per region (spatial unit) (Fig. 1). Currently ESPRES has two modules, designed to consider two different water pressures. The Quantity module focuses on water stress, and comprises five sectors contributing to alterations of water quantity, namely domestic, energy, livestock, irrigation and industrial sectors. The Quality module tackles nutrient (specifically nitrogen) pollution; in this module four sectors are defined as main responsible for nitrogen emissions, namely nitrogen from manure fertilization, from mineral fertilization, from point sources, and from scattered dwellings.

The spatial units are the administrative regions of the basin. Currently, ESPRES considers European Nuts2 regions (https://en.wikipedia.org/wiki/Category:NUTS_2_statistical_regions_of_the_European_Union). These are regarded as the relevant units for environmental planning, because at this level political decisions can be made independently. It is also possible to select a higher aggregation level. At the moment, ESPRES includes four European basins: Adige (Italy), Ebro (Spain), Evrotas (Greece), and Sava (Slovenia, Croatia, Bosnia and Herzegovina, Montenegro, Albania and Serbia), which are case studies of the Globaqua project (Navarro-Ortega et al., 2015; GLOBAQUA, 2018a, GLOBAQUA, 2018b). In the future, ESPRES will be extended to all European basins. The four basins are affected by water scarcity either due to climatic or societal reasons, but anthropogenic stressors acting on the freshwater network are different (Lutz et al., 2016). The number of administrative regions of a river basin depends on its size and geography: one in the Evrotas, four in the Adige, 10 in the Sava, and 11 in the Ebro. In this study, nutrient pollution in the transboundary Sava River Basin is taken as an example to illustrate the tool. Tutorials that extend analysis to water stress and to other basins are available on the ESPRES pages (www.espres.eu) and on the e-learning platform OpenTea (http://www.opentea.eu/en/e-learning/courses-Managing-the-effects-of-multiple-stressors-on-aquatic-ecosystems-under-water-scarcity.25/).

A management strategy comprises a number of variables equal to the number of administrative regions times the number of sectors contributing to the pressure. For the Sava example, a strategy for nutrient pollution reduction contains 40 decision variables (four sectors times ten regions). For each variable, an independent reduction rate is set (Fig. 1). Each strategy defined in the *decision space* (represented by a table of pressure reduction rates per sector and per region in Fig. 1) corresponds to a result in the *objective space*, i.e. a combination of one environmental outcome for a given effort (represented by a point in Fig. 1).

### 2.2 The environmental outcome

The new condition of the aquatic environment achieved by a strategy is called in ESPRES the Environmental outcome. The environmental performance of a strategy depends on many factors, including the biophysical characteristics of the region, the local and upstream pressures, the initial condition and the extent of the changes. ESPRES uses European scale data and models to assess environmental outcomes (pressure reduction) of the management strategies.

In the Quantity module the environmental objective is evaluated with the water exploitation index (WEI), which is an indicator of water stress (Alcamo et al., 2000). WEI is defined as the ratio between the total annual water abstraction and the long-term mean annual freshwater availability (UNECE, 2006). A WEI threshold of 20% indicates a water stressed region; a WEI higher than 40% indicates severe water stress (Raskin et al., 1997). Details on how WEI is calculated in ESPRES are in the Supplementary material.

In the Quality module, the environmental objective is quantified with the mean annual Total Nitrogen concentration (TN, mg/L). Values of TN lower than 1.5 mg/L indicates good quality, while values higher than 20 mg/L indicates very bad quality (class I and class V, ICPDR water quality classification, annex). TN is estimated with the model GREEN (Grizzetti et al., 2012), which is a European conceptual model that considers nitrogen emissions from diffuse and point sources.

The environmental models work at hydrologic catchment scale (average size of 7 km^2^ for the Quality module). Each catchment belongs to a given administrative region. The environmental impact of management strategies is computed at the catchment level, but the results are aggregated at the administrative region level and at the basin scale. The aggregated metric at the basin scale provides the strategy environmental outcome, i.e. the value of the first dimension in the *objective space*.

### 2.3 The effort objective

The effort objective summarizes the difficulty to tackle the environmental pressure at the basin scale. The effort to reduce a pressure in an administrative region or in a specific sector is defined by the user, who must express the difficulty of reducing a pressure by a given percentage considering the technical feasibility of potential solutions, the financial costs, and the socio-political acceptability of solutions. The effort thus goes beyond the mere consideration of monetary cost, and allows considering technical know-how as well as political perceptions, which may be important especially in basins that extends across administrative regions or even countries. For example, for the nutrient pollution case, the effort should consider how difficult would be to reduce mineral fertilization in agricultural areas rather than improving wastewater treatment or reduce emissions from scattered dwellings in each region.

**Fig. 2:**
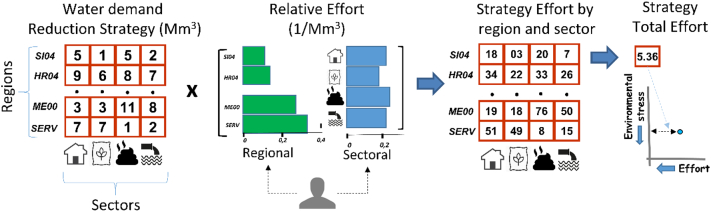
Evaluating the effort for one strategy to reduce nutrients concentration. The relative effort to reduce pressure in each region and sector is set by the user. The strategy total effort is the sum of the efforts of all regions and sectors.

The relative effort to reduce pressures is defined by the user for all sectors and all administrative regions. Once relative efforts are assigned, ESPRES computes the total effort for each strategy as the sum of all the efforts in each region and sector weighted by the reduction rate (Fig. 2), thus providing the value of the second dimension in the *objective space* (Fig. 1).

### 2.4 Architecture

**Fig. 3:**
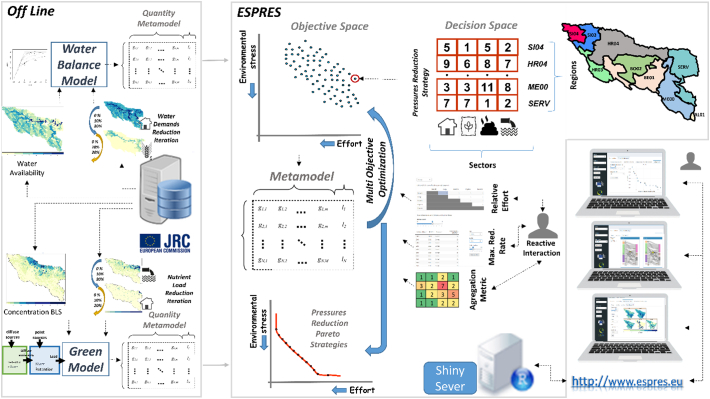
The overall architecture of ESPRES. ESPRES is driven by metamodels informed by biophysical models. The metamodels compute outputs for any input combination with an additive linear model based on pre-generated input and outputs. The user interacts with ESPRES through a web-based visualization toolkit.

ESPRES includes four main components (Fig. 3): (1) A geodatabase storing river basin data (including, catchment network structure, baseline conditions, current environmental pressures); (2) A multi-objective optimization (MOO) engine designed to search the regional and sectoral reduction effort strategy; (3) The meta-models to simulate the pressures reduction effort and the environmental outcome under different management strategies; (4) A user-friendly web Graphical User Interface to run the analysis.

The core of the tool is the MOO engine that identifies trade-offs between environmental outcomes and effort, and efficient strategies in the objective space. ESPRES applies multi objective genetic algorithm (GA) as optimization technique to find efficient management strategies. GA are population-based, stochastic search techniques, widely used as efficient global optimizer. However, optimization methods usually require numerous evaluations of candidate solutions. For many real world problems, the evaluation of a single function/model is computationally time-consuming and hence often practically prohibitive. The solution for using GA in complex applications lies in the use of metamodels that replace the original functions by quicker approximate functions (Razavi et al., 2012), which abate computation time substantially (Bhattacharya, 2013).

The original environmental models were not ingested directly but employed to create two metamodels (one for the water quantity and one for water quality). The metamodel is like a multiple linear regression where the effect of the concurrent application of multiple stressors in a basin is calculated from the sum of individual contributions of each model spatial unit. In practical terms, the metamodel is a matrix with the environmental stress reduction in each sub-basin brought by a 1% reduction of pressure in each pressure driver. The matrices have been calculated running the environmental models applying stepwise reductions of pressures on individual (regional and sectorial) units (Fig. 3).

The web interface provides a point of access to search for efficient pressure reduction strategies reflecting the stakeholder (i.e. the web-tool user) perception of entailed effort. The tool has functionalities to set the effort, explore the baseline situation in relation to environmental pressures and water status, analyse and visualize changes due to management strategies through graphs and maps. The tool applies a management reduction strategy, assesses the strategy environmental outcome (new water status), and compares it with the current basin condition (Baseline Scenario, BLS) or another solution.

### 2.5 Software implementation

ESPRES is implemented using R (R Core Team, 2014), an open-source software for statistical computing. The client/server application is coded in Shiny (RStudio, Inc., 2014; Chang et al., 2019) that provides a standardized, interactive interface for visualizing, exploring and analyzing pressures reduction management strategies. Shiny framework uses a reactive programming model and provides an automatic binding reaction between inputs, outputs and widgets to build dynamic web applications. Reactivity lets the app instantly update itself whenever the user makes a change. It is also easy to customize the overall appearance using JavaScript, CSS stylesheet and HTML code.

The MOO engine is implemented using packages ‘mco’ (Multiple Criteria Optimization Algorithms and Related Functions; Mersmann et al., 2014) and ‘*nsga2R’* (Elitist Non-dominated Sorting Genetic Algorithm, Tsou, 2013). Results of ESPRES are displayed as tables with ‘*DT’* (Xie et al., 2019) and figures with ‘*plotly’* (Sievert et al., 2019) and ggplot2 (Wickham et al., 2019). Other figures and maps are created with *sp* (Classes and Methods for Spatial Data; Pebesma et al., 2018), ‘*maptools’* (Bivand et al., 2019b), ‘*rgdal’* (Bivand et al., 2019a), and *rgeos* (Bivand et al., 2019c). Other R packages used in the source code comprise ‘*ncdf4’* (Pierce, 2019) to read stored NetCDF scenario datasets; ‘*plyr’* (Wickham, 2016), ‘*reshape’* (Wickham, 2018), ‘*reshape2’* (Wickham, 2018) to manipulate datasets; ‘*fields’* (Nychka et al., 2019), ‘*RColorBrewer’* (Neuwirth, 2014), ‘*classInt’* (Roger et al., 2019) and ‘*networkD3’* (Allaire et al., 2017) for visualization purposes. Finally, ‘*shinydashboard’* (Chang and Borges Ribeiro, 2018), ‘*shinyjs’* (Attali, 2018) and ‘*shinyWidgets’* (Perrier et al., 2018) provide additional functionality and interactivity to Shiny applications.

## 3 Critical steps of the pressure reduction analysis

**Fig. 4:**
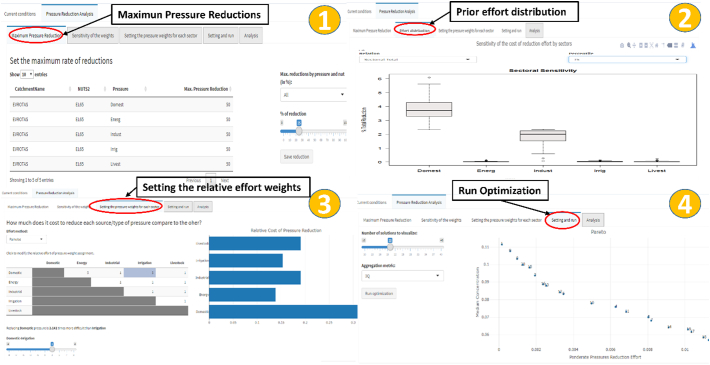
The four consecutive interactive steps to configure the pressure reduction analysis: 1) setting maxima pressure reductions; 2) looking at prior effort distribution; 3) setting the relative effort weights; 4) setting optimization parameter.

The Pressure Reduction Analysis in ESPRES is the optimization process to search for efficient strategies that defines trade-offs between environmental outcome and effort. The process is controlled through a number of user choices; in particular, there are four consecutive steps to configure the analysis (Fig. 4). These interactions enable the user to tailor the analysis to his/her perception.

The first step is the definition of the maximum possible reduction that is allowed for each sector in each administrative region. This defines the limits of the search space; the optimization process will not look for efficient solutions beyond them. These maxima are set by the user according to physical or socio-political context.

The second step is the prior appraisal of solutions for equal effort distribution. This step is purely informative and does not affect the pressure reduction analysis, but provides a first overview of what can be done and where. This step offers an initial reference of optimal distribution of the pressure reductions between sectors and administrative regions when all efforts are set equal. It is informed by one hundred previous optimization executions done for three prior references level of pressure reduction, i.e. reducing the median environmental metric at 90%, 75% and 50% of its current value. Boxplot graphs display the distribution of the effort shares between sectors and regions (see more details in the additional material).

The third step consists of assigning effort weights to each sector and region, thus tailoring the analysis according to the user's perception. By default all weights are equal to 1 (equal effort in all cases). When reducing a pressure by acting on one sector (and/or in one region) implies a different effort than acting elsewhere, the user should set relative weights that reflect this difference. Weights can be assigned in ESPRES directly or by pairwise comparison. In the pairwise comparison method, the user consider two options at once, and indicates how much more difficult would be to tackle the same pressure in a certain region (or in a certain sector) than in another. The user can press one by one in the boxes of the pairwise comparison table and move the slider to assign weights that considers reflecting the actual situation of the basin. ESPRES automatically displays the relative weights by sector/regions and provide warnings in case of inconsistent assignment of weights. The advantage of pairwise comparison over direct assignment is that the user considers only two element at once over which to express its judgment.

The fourth and final step is the execution of the optimization process. The user must make two important choices: (i) setting the number of solutions that the Pareto front should contain, and (ii) selecting the aggregation metric with which the optimizer shall evaluate the environmental objective of the strategies. The aggregation metric of the environmental outcome has a great influence on the optimization process and on its resulting efficient strategies. In ESPRES, five aggregation operators are available, namely the mean, median, third quartile, maximum, and threshold, which is the sum of values above a given threshold set by the user. When the mean metric is selected, optimal strategies typically applies more effort in regions or sectors where the same effort produces greater local improvement (even if there are no environmental issues), whereas when the maximum or the threshold are selected, the optimization tends to focus effort in regions where environmental problems are greater.

Clearly, different stakeholders may have different opinions about the maximum reduction that could be realistically achieved (step 1), or about the effort entailed in reducing pressures in given sectors or regions (step 3). Since these choices are crucial aspects of the analysis, the tool was designed to recognize them explicitly.

The tool is completed with means to identify and compare efficient management strategies. ESPRES enables local stakeholders and regional experts to re-do the analysis formulating different weights and maximum reduction thresholds that reflect their judgment. This may help in a process of negotiation and brings transparency and awareness of potential limits of management plans.

## 4 Application of ESPRES to the Sava river basin

The analysis of nutrient pollution in the Sava river basin is given as an example to illustrate how ESPRES can be used to examine the environmental state of a basin in relation to the most important acting pressures, and to showcase the importance of the tool interactive steps to identify efficient reduction strategies.

The Sava River (945 km) is the largest tributary of the Danube River. The basin extends over 9,771,320 km^2^ across Slovenia, Croatia, Bosnia and Herzegovina, Serbia, Montenegro, and Albania (ISRBC, 2009). The climate varies across the basin from alpine to moderate continental depending on orography and influence of the sea. Mean annual precipitation ranges from about 1100 mm in the alpine area of Slovenia to about 650 mm in the Serbian plains. Cultivated land covers 23.2% of the basin, pasture 6.7%, boreal forest 1.5%, mixed forest 31.7%, and deciduous forest 36.1% (Levi et al., 2015). The population in the Sava basin is around 8.2 million (ISRBC, 2009).

**Fig. 5:**
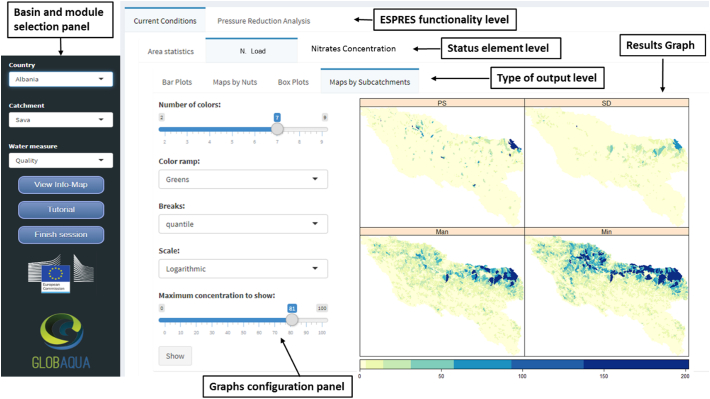
Screenshot of ESPRES current conditions tab functionalities in the quality module; the maps show the subcatchment distribution of the four sectorial total nitrogen sources in the Sava basin: PS = point sources; SD = scattered Dwellings; Man = Manure application; Min = mineral fertilization.

Once ESPRES interface is loaded into the browser (www.espres.eu), the user selects a basin and the type of analysis (quantitative or qualitative; Fig. 5). Before looking for optimal management strategies, it is convenient to examine the current state of the basin. The current conditions tabs (Fig. 5) provide options to explore the state of the basin through maps, graphs and tables. By browsing these options, it is possible to explore what type of pressures are dominant, where the main problems are located, and which pressure sectors are causing the highest impact.

**Fig. 6:**
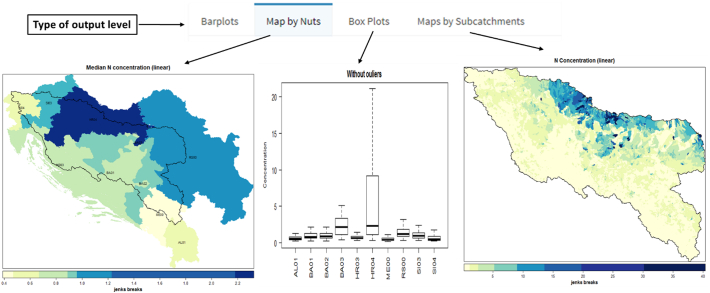
Three types of visualization outputs in ESPRES (left to right): map by administrative regions, boxplots and map by subcatchment. In the example, Total Nitrogen concentration in the Sava basin is shown. The user can choose color scale, scale type, and the breaks distribution for map visualization.

In the output tab level, the environmental indicators are shown in graphs and maps (Fig. 6) and can be browsed by regions or by spatial model units (subcatchments).

**Fig. 7:**
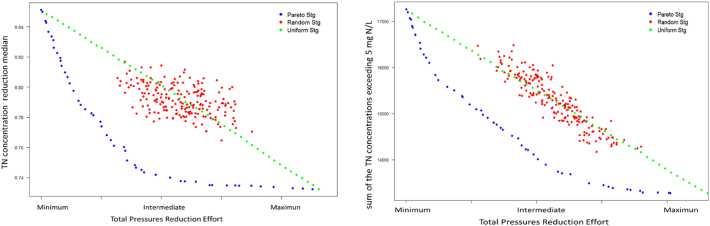
Strategies Objective Space showing effort vs pressure reduction: Pareto front (blue), uniform distribution of the applied effort (green), random reduction strategies (red). Both plots show results coming from the quality module applied in the Sava basin assigning equal effort to all sectors or regions. Aggregation metric to assess the quality stress are the median of the TN concentration in each subcatchment (left) and the threshold (sum of the value of the TN concentrations exceeding 5 mg N/L, right). (For interpretation of the references to color in this figure legend, the reader is referred to the web version of this article.)

After going through the four interactive steps of the Pressure Reduction Analysis optimization, ESPRES interface shows a Pareto front of efficient strategies. For example, Fig. 7 shows two Pareto fronts for the Sava basin. In both cases, maximum reduction was set to 50% (step 1), and effort weights were set all equal (step 3). The two optimization runs differ for the environmental aggregation metric, which was either the median of Total Nitrogen (TN, mg N/L; Fig. 7 left), or the threshold (i.e the sum of TN concentrations exceeding 5 mg/L; Fig. 7 right). The plots are limited by the current situation (top left point, zero reduction effort) and the maximum improvement that could be achieved when applying the maximum pressure reduction (right lower point). Efficient strategies belonging to the Pareto front are shown in blue, whereas random generated strategies are shown in red, and strategies with uniform distribution of effort (i.e., applying same reduction rate in all regions and sector) are in green. In both analysis, efficient strategies (blue dots) could provide environmental improvements similar to those obtained with a uniform distribution of effort, but with a considerable reduction of the total effort.

**Fig. 8:**
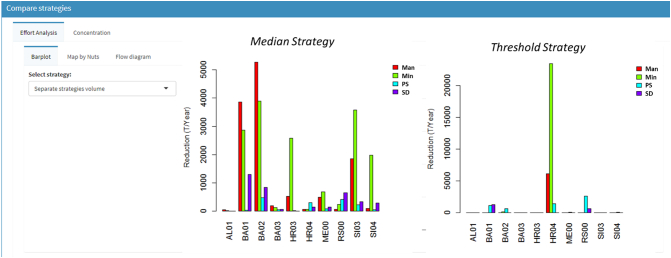
Barplot comparing the distribution of efforts of two optimal strategies of similar total efforts to reduce nutrient emissions in the Sava basin. The strategy on the left targeting a reduction of median TN concentration applies more effort in upstream regions, where environmental pressure is lower; the strategy on the right, targeting reduction of pollution in reaches above a threshold of 5 mg N/L, focused effort in the most polluted region (HR04).

The user can then select strategies (points) from the Pareto front that are considered of interest to examine them more carefully, e.g. looking at how the pressure reduction is distributed and what environmental state is achieved, and comparing them with each other or with the current state. In ESPRES pressure reductions of a strategy can be displayed through barplots, boxplots, maps, and flow diagrams in terms of rate of change or absolute quantities, and aggregated by sectors or region. For example, Fig. 8 shows the bar plot diagrams of two strategies of similar total effort for total nitrogen reduction in the Sava basin, extracted by the Pareto fronts shown in Fig. 7. While the total efforts of the two strategies are similar, the distribution of efforts by regions is very different: the median strategy targeted effort in the upstream areas (Fig. 8, left) while the threshold strategy focused most effort in Croatian region HR04 (right).

**Fig. 9:**
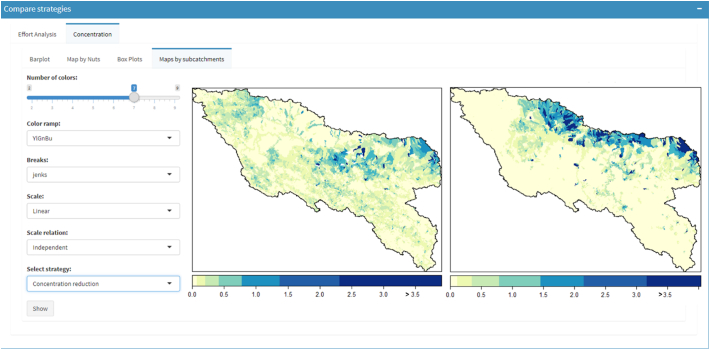
Screenshot of the Total Nitrogen concentration reduction (related with the current situation) in the Sava basin for two different strategies of similar total effort (Fig. 8 strategies).

As a consequence of the different distribution of efforts, the reduction of TN concentration achieved by the strategies differ notably too (Fig. 9).

## 5 Discussion and conclusions

Science-policy integration is one of the most complex challenges that scientific and policy-making communities are facing; it involves communication, knowledge sharing, and exchanges among a wide range of disciplines and actors (Quevauviller et al., 2005). In many instances, poor communication and interactions lead to scientific outputs not being used or even known by policy-makers, and to policy research needs not being addressed timely by the scientific communities.

Enabling an open and transparent debate in river basin management is not trivial. Typically, the complexity of environmental models circumscribes them to scientific laboratories and academic computer clusters. Often, scenario analysis is confined to a small number of pre-defined options, which may or may not have been discussed with major stakeholders. As a result, decision-makers may interpret model outputs either as hard evidence in support of specific policies or, at the opposite side, dismiss them as inconclusive when a distrust for incomprehensible ‘black boxes’ prevails.

The information provided by environmental models into policy formulation must be channelled through accessible media that ideally would empower stakeholders, allowing exploration of issues and potential alternatives. Recent technological innovations in networking and computing (among which those that underpin interactive Web-based tools) bring a new generation of platforms that allow stakeholders or citizens to interact with scientific models more directly. Such interactivity has the potential to turn the typical top-down flow of information from scientists to users into a more engaging and empowering process. It opens perspectives to speed up the dissemination of environmental information to a larger community of users, to harvest feedback, and to widen analysis of model predictions from different angles. However, this evolution comes with the challenge of communicating modelled results in such a way that they can be interpreted and used correctly (Buytaert et al., 2012).

ESPRES was born with the objective of responding to all these problems. The DSS helps multi-criteria river basin analyses of drivers, pressures, status, impact, and responses to identify most efficient investments to strike a balance between benefits to nature and to people. ESPRES promotes hands-on exploration of what can be done and where; it can be used to understand current conditions and what can be potentially achieved and how, to visualize and interpret trade-offs, examining options, helping negotiations, and enhancing management transparency.

As an open-access exploratory web tool that does not require installation, it is very flexible and allows going beyond pre-cooked scenarios. Through a user-friendly interface and in a few seconds, it offers the opportunity to identify efficient strategies and analyse alternatives for what the user considers being a reasonable effort. Among efficient strategies that require a similar effort there may be common patterns of actions (i.e. consistent reductions in some sectors of regions) or conversely, be completely different. Managers can thus better consider what could be achieved with each strategy, and select the best course of action. All this helps taking informed decisions and planning policies that are efficient to achieve environmental goals and that are affordable from an economic, social and political point of view.

Expressing the socio-economic objective in terms of generic effort and relative weights allows regional experts to include considerations of local difficulties in applying pressure reductions in a given region or sector. This functionality enables to explore where the effort should be better applied in a transparent way, and to appreciate differences in outcomes of strategies locally and at the basin scale, ultimately helping justify the course of actions taken by decision-makers. In addition, it can enable to find a consensus on the proposed values for weights among groups of experts/decision-makers, reducing the subjectivity of the opinion of a single expert (Cook and Seiford, 1978).

The tool can also be used for educational case studies as it shows (i) the importance of clearly defining the environmental goal, and (ii) that by choosing carefully where to concentrate effort, large improvements towards environmental targets can be achieved. An important feature of ESPRES is the possibility to select between different aggregation metrics for the calculation of the environmental outcome. These metrics reflect different management perspectives, for example if the decisions are to improve average conditions across a basin or reduce pollution in hotspots.

ESPRES has been developed on the basis of data and models available for all European basins. Although at the moment it is only functional for four pilot basins, it will soon be expanded to include all European basins. A limitation of the current version is that the system considers the multiple pressures related to water stress on the one hand, and nutrient pollution on the other, but not both pressures simultaneously.

ESPRES can help stakeholders exploring management options for reducing the impact of anthropogenic pressures in European basins. The analysis may be used to identify combinations of pressure reduction actions and to guide effective design of programmes of measures that needs to be verified by local feasibility studies. Overall ESPRES can contribute to the improvement of river basin management planning and the design of programmes of measures in the European Union.

## CRediT authorship contribution statement

**Angel Udias:** Conceptualization, Methodology, Software, Visualization, Data curation, Writing - original draft. **Alberto Pistocchi:** Supervision, Conceptualization, Project administration, Methodology, Writing - review & editing. **Olga Vigiak:** Conceptualization, Methodology, Investigation, Writing - review & editing. **Bruna Grizzetti:** Conceptualization, Methodology, Investigation, Writing - review & editing. **Faycal Bouraoui:** Conceptualization, Methodology, Validation. **Cesar Alfaro:**Software, Data curation, Visualization.

## Declaration of competing interest

The authors declare that they have no known competing financial interests or personal relationships that could have appeared to influence the work reported in this paper.

## Appendix A Supplementary data

Additional Data and Environmental Model Details Image 1

## References

[bb0005] Alcamo J. (2008). The SAS approach : combining qualitative and quantitative knowledge in environmental scenarios. Environ. Futur. Pract. Environ. Scenar. Anal..

[bb0010] Alcamo J., Henrich T., Rösch T. (2000). World Water in 2025—Global Modeling and Scenario Analysis for the World Commission on Water for the 21st Century. ReportA0002.

[bb0015] Allaire J.J., Ellis P, Gandrud C, Kuo K, Lewis B.W., Owen J, Russell K, Rogers J, Sese C, Yetman CJ. 2017. *networkD3*: D3 JavaScript network graphs from R. R package version 0.4, URL http://CRAN.R-project.org/package= networkD3.

[bb0020] Attali D., 2018. *shinyjs*: easily improve the user experience of your shiny apps in seconds. R package version 1.0 URL http://CRAN.R-project.org/package= shinyjs.

[bb0025] Berntsen P.B., Trutnevyte E. (2017). Ensuring diversity of national energy scenarios: Bottomup energy system model with modeling to generate alternatives. Energy.

[bb0030] Bhattacharya M. (2013). Expensive optimisation: a metaheuristics perspective. Int. J. Adv. Comput. Sci. Appl..

[bb0035] Bivand R, Keitt T, Rowlingson B, Pebesma E, Sumner M, Hijmans R, Rouault E, Warmerdam F, Ooms J, Rundel C., 2019a. *rgdal*: bindings for the 'Geospatial' data abstraction library. R package version 1.4-4, URL http://CRAN.R-project.org/package= rgdal.

[bb0040] Bivand R, Lewin-Koh N, Pebesma E, Archer E, Baddeley A, Bearman N, Bibiko H, Brey S, Callahan J, Carrillo G, Dray S, Forrest D, Friendly M, Giraudoux P, Golicher D, Gómez Rubio V, Hausmann P, Ove Hufthammer K, Jagger T, Johnson K, Luque S, MacQueen D, Niccolai A, Pebesma E, Perpiñán O, Plunkett E, Short T, Snow G, Stabler B, Stokely M, Turner R., 2019b. *maptools*: tools for handling spatial object. R package version 0.9-5, URL http://CRAN.R-project.org/package= maptools.

[bb0045] Bivand R., Rundel C., Pebesma E., Stuetz R., Ove Hufthammer K., Giraudoux P., Davis M., Santilli S., 2019c. *rgeos*: Interface to geometry engine - open source ('GEOS') R package version 0.4-3, URL http://CRAN.R-project.org/package= rgeos.

[bb0050] Booty W.G., Lam D.C.L., Wong I.W.S., Siconolfi P. (2001). Design and implementation of an environmental decision support system. Environ. Model Softw..

[bb0060] Buytaert W., Baez S., Bustamante M., Dewulf A. (2012). Web-based environmental simulation: bridging the gap between scientific modeling and decision-making. Environmental Science & Technology.

[bb0070] Chang W., Borges Ribeiro B., 2018. *shinydashboard*: create dashboards with 'Shiny'. R package version 0.7.1 URL http://CRAN.R-project.org/package= shinydashboard.

[bb0075] Chang, W., Cheng, J., Allaire, J. J., Xie, Y., and McPherson, J., 2019. Shiny: Web Application Framework for R. R package version 1.3.2, URL http://CRAN.R-project.org/package=shiny.

[bb0080] Cook W., Seiford L. (1978). Priory ranking and consensus formation. Manag. Sci..

[bb0085] Coors A., De Meester L. (2008). 2008. Synergistic, antagonistic and additive effects of multiple stressors: predation threat, parasitism and pesticide exposure in Daphnia magna. J. Appl. Ecol..

[bb0090] De Carolis J.F., Babaee S., Li B., Kanungo S. (2016). Modelling to generate alternatives with an energy system optimization model. Environ. Model. Softw..

[bb0105] European Parliament and Council (2000). Directive 2000/60/EC establishing a framework for community action in the field of water policy. Official Journal of the European Union L.

[bb0115] Giupponi C., Sgobbi A. (2013). Decision support systems for water resources management in developing countries: learning from experiences in Africa. Water.

[bb0120] GLOBAQUA. 2018a. Policy recommendations, Globaqua Deliverable D012. Globaqua project report D11.12. http://www.globaqua-project.eu/files/repository/20180720131147_20180205110856_GLOBAQUADeliverable012(D11.12).pdf.

[bb0125] GLOBAQUA (2018). Final integrated models at the basin scale, Globaqua deliverable D016. Globaqua project report D9.16. http://www.globaqua-project.eu/en/documents/showcategory/&cat=press.

[bb0130] Grizzetti B., Bouraoui F., Aloe A. (2012). Changes of nitrogen and phosphorus loads to European seas. Glob. Chang. Biol..

[bb0135] Guivarch C., Lempert R., Trutnevyte E. (2017). Scenario techniques for energy and environmental research: an overview of recent developments to broaden the capacity to deal with complexity and uncertainty. Environ. Model. Softw..

[bb0140] Harou J.J., Pulido-Velazquez M., Rosenberg D.E., Medellín-Azuara J., Lund J.R., Howitt R.E. (2009). Hydro-economic models: concepts, design, applications, and future prospects. J. Hydrol..

[bb0145] Hashemi F., Olesen J.E., Dalgaard T., Børgesen C.D. (2016). Review of scenario analyses to reduce agricultural nitrogen and phosphorus loading to the aquatic environment. Sci. Total Environ..

[bb0150] Holmes J., Clark R. (2008). Enhancing the use of science in environmental policy-making and regulation. Environ. Sci. Pol..

[bb0155] ISRBC (2009). The Sava River Basin Analysis Report.

[bb0160] Kalogianni E., Vourka A., Karaouzas I., Vardakas L., Laschou S., Skoulikidis N.T. (2017). Combined effects of water stress and pollution on macroinvertebrate and fish assemblages in a Mediterranean intermittent river. Sci. Total Environ..

[bb0165] Kunz W., Rittel H. (1970). Issues as Elements of Information Systems. Working Paper No. 131.

[bb0170] Levi L., Jaramillo F., Andričević R., Destouni G. (2015). Hydroclimatic changes and drivers in the Sava River catchment and comparison with Swedish catchments. Ambio.

[bb0175] Lutz S.R., Mallucci S., Diamantini E., Majone B., Bellin A., Merz R. (2016). Hydroclimatic and water quality trends across three Mediterranean river basins. Sci. Total Environ..

[bb0180] Maier H.R., Kapelan J., Kasprzyk J., Kollat L.S., Matotte G.C., Dandya E., Keedwell A., Marchi A., Ostfeld D., Savic Solomatine D.P., Vrugt J.A., Zecchin A.C., Minsker B.S., Barbour E.J., Kuczera G., Pasha F., Castelletti A., Giuliani M., Reed P.M. (2014). Evolutionary algorithms and other metaheuristics in water resources: current status, research challenges and future directions. Environ. Model. Softw..

[bb0185] Matthies M., Giupponi C., Ostendorf B. (2007). Environmental decision support systems: current issues, methods and tools. Environ. Model. Softw..

[bb0190] McInerny G.J., Chen M., Freeman R., Gavaghan D., Meyer M., Rowland F., Spiegelhalter D.J., Stefaner M., Tessarolo G., Hortal J. (2014). Information visualisation for science and policy: engaging users and avoiding bias. Trends Ecol. Evol..

[bb0195] Mersmann O., Trautmann H., Steuer D., Bischl B., Deb K. (2014). *mco*: multiple criteria optimization algorithms and related function. R package version 1.0-15.1. http://CRAN.R-project.org/package=mco.

[bb0205] Navarro-Ortega, A., Acuna, V., Bellin, A., Burek, P., Cassiani, G., Choukr-Allah, R., Doledec, S., Elosegi, A., Ferrari, F., Ginebreda, A., Grathwohl, P., Jones, C., Ker rault, P., Kok, K., Koundouri, P., Ludwig, R.P., Merz, R., Milacic, R., Munoz, I., Nikulin, G., Paniconi, C., Paunovic, M., Petrovic, M., sabater, L., Sabater, S., Skoulikidis, N. T., Slob, A., Teutsch, G., Voulvoulis, N., Barcelo’, D., 2015. Managing the effects of multiple stressors on aquatic ecosystems under water scarcity. Sci. Total Environ. 503-404, 3–9. 10.1016/j.scitotenv.2014.06.081 PMC4236898 25005236

[bb0210] Neuwirth E., 2014. *RColorBrewer*: ColorBrewer palettes. R package version 1.1-2, URL http://CRAN.R-project.org/package= RColorBrewer.

[bb0215] Nychka D., Furrer R., Paige J., Sain S., 2019. *fields*: tools for spatial data. R package version 9.8-3, URL http://CRAN.R-project.org/package= fields.

[bb0220] Ormerod S.J., Dobson M., Hildrew A.G., Townsend C.R. (2010). Multiple stressors in freshwater ecosystems. Freshw. Biol..

[bb0225] Pebesma E, Bivand R, Rowlingson B, Gomez-Rubio V, Hijmans R, Sumner M, MacQueen D, Lemon J, O'Brien J, O'Rourke J., 2018. *sp*: classes and methods for spatial data. R package version 1.3-1, URL http://CRAN.R-project.org/package= sp.

[bb0230] Perrier V., Meyer F, Granjon D., 2018. *shinyWidgets*: custom inputs widgets for shiny R package version 0.4.8 URL http://CRAN.R-project.org/package= shinyWidgets.

[bb0235] Pierce D., 2019. *ncdf4*: Interface to Unidata netCDF (version 4 or earlier) format data files. R package version 1.16.1, URL http://CRAN.R-project.org/package= ncdf4.

[bb0240] Pistocchi A., Udias A., Grizzetti B., Gelati E., Kondouri P., Ludwig R., Papandreou A., Souliotis I. (2017). An integrated assessment framework for the analysis of multiple pressures in aquatic ecosystems and the appraisal of management options. Sci. Total Environ..

[bb0250] Quevauviller P., Balabanis P., Fragakis C., Weydert M., Oliver M., Kaschl A., Arnold G., Kroll A., Galbiati L., Zaldivar J.M., Bidoglio G. (2005). Science-policy integration needs in support of the implementation of the EU Water Framework Directive. Environ. Sci. Pol..

[bb0255] R Core Team (2014). R: a language and environment for statistical computing. http://www.r-project.org.

[bb0260] Raskin P., Gleick P.H., Kirshen P., Pontius G., Strzepek K. (1997). Comprehensive Assessment of the Freshwater Resources of the World.

[bb0265] Razavi S., Tolson B.A., Burn D.H. (2012). Review of surrogate modeling in water resources. Water Resour. Res..

[bb0270] Rittel H., Webber M. (1973). Dilemmas in a general theory of planning. Policy. Sci..

[bb0275] Roger B, Ono H, Dunlap R, Stigler M., 2019 *classInt*: choose Univariate class intervals. R package version 0.3-3, URL http://CRAN.R-project.org/package= classInt.

[bb0280] RStudio, Inc, 2014. Shiny: web application framework for R. R package version 0.10.1. http://CRAN.R-project.org/package¼shiny.

[bb0285] Sandink D., Simonovic S.P., Schardong A., Srivastav R. (2016). A decision support system for updating and incorporating climate change impacts into rainfall intensity-durationfrequency curves: review of the stakeholder involvement process. Environ. Model. Softw..

[bb0290] Sievert C., Parmer C., Hocking T., Chamberlain S, Ram K, Corvellec M., Despouy P., 2019. *plotly*: create interactive web graphics via 'plotly.js'. R package version 4.9, URL http://CRAN.R-project.org/package= plotly .

[bb0295] Sivapalan M., Savenije H., Bloeschl G. (2012). Sociohydrology: a new science of people and water. Hydrol. Process..

[bb0300] Stevenson R.J., Sabater S. (2010). Understangind global change in river ecosystems: science to support policy in a changing worl. Hydrobiologia.

[bb0305] Tsou C. (2013). *nsga2R*: elitist non-dominated sorting genetic algorithm. R package version 1.0. http://CRAN.R-project.org/package=nsga2R.

[bb0310] Udias A., Malagò A., Pastori M., Vigiak O., Reynaud A., Elorza F.J., Bouraoui F. (2016). Identifying efficient nitrate reduction strategies in the Upper Danube. Water.

[bb0315] Udias A., Pastori M., Malago A., Vigiak O., Nikolaidis N.P., Bouraoui F. (2018). Identifying efficient agricultural irrigation strategies in Crete. Sci. Total Environ..

[bb0320] United Nations. Economic Commission for Europe (UNECE) (2006). Environment Indicators and Indicator-Based Assessment Reports: Eastern Europe, Caucasus and Central Asia (ECE/CEP/140).

[bb0335] Voinov A., Kolagani N., McCall M.K., Glynn P.D., Kragt M.E., Ostermann F.O., Pierce S.A., Ramu P. (2016). Modelling with stakeholders - next generation. Environ. Model. Softw..

[bb0340] Vörösmarty C.J., McIntyre P.B., Gessner M.O., Dudgeon D., Prusevich A., Green P., Glidden S. (2010). Bunn S. E., , Sullivan C.A., Reidy Liermann C, Davies P. M., 2010. Global threats to human water security and river biodiversity. Nature.

[bb0345] Wickham H., 2016. *plyr*: tools for splitting, applying and combining data. R package version 1.8.4, URL http://CRAN.R-project.org/package= ncdf4.

[bb0355] Wickham H., 2018. *reshape*: flexibly reshape data. R package version 0.8.8, URL http://CRAN.R-project.org/package= reshape.

[bb0360] Wickham H., Chang W., Henry L., Lin Pedersen T., Takahashi K., Wilke C., Woo K., 2019. *ggplot2*: create elegant data visualisations using the grammar of graphics. R package version 3.1.1, URL http://CRAN.R-project.org/package= ggplot2.

[bb0365] Xie Y., Cheng J., Tan X., Allaire J.J., Girlich M., Freedman G., Rauh J. (2019). *DT*: a wrapper of the JavaScript library 'DataTables'. R package version 0.6. http://CRAN.R-project.org/package=DT.

[bb0370] Zalewski M., Janauer G.A., Jolankai G. (1997). Ecohydrology. A new paradigm for the sustainable use of aquatic resources. UNESCO IHP Technical Document in Hydrology No. 7.; IHP - V Projects 2.3/2.4.

