# Supplementary material for: ESPRES: A web application for interactive analysis of multiple pressures in aquatic ecosystems
Source: Sci Total Environ. 2020 Nov 20;744:140792. doi: 10.1016/j.scitotenv.2020.140792 (PMC7511691; doi:10.1016/j.scitotenv.2020.140792)

# Additional Material

Sava nitrogen inputs

*Table AM1. Nitrogen inputs applied in the Sava river basin by region and type of source. (Man: Manure; Min: Mineral: PS: Point Sources; SD: Scattered Dwellings)*

| **Regigon** | **NUTS ID** | **N subcatch** | **Area(km^2^)** | **Nitrogen Input (t/y)** |  |  |  |  |
| --- | --- | --- | --- | --- | --- | --- | --- | --- |
|  |  |  |  | **Man** | **Min** | **PS** | **SD** | **%** |
| Veri | AL01 | 100 | 136 | 103.8 | 27.0 | 0.0 | 5.0 | 0.07 |
| Bosnei Hercegovine | BA01 | 5827 | 16651 | 7745.7 | 5746.4 | 2253.1 | 2619.1 | **9.94** |
| Republika Srpska | BA02 | 5180 | 20611 | 10696.5 | 7805.3 | 1371.2 | 1774.2 | **11.72** |
| Brčko Distrikt | BA03 | 34 | 471 | 403.5 | 253.9 | 109.9 | 137.0 | 0.49 |
| Jadranska Hrvatska | HR03 | 1949 | 6417 | 1057.0 | 5155.2 | 43.2 | 2.9 | 3.39 |
| Kontinentalna Hrvatska | HR04 | 2448 | 22718 | 14219.9 | **58830.5** | 3139.3 | 294.8 | **41.40** |
| Montenegro | ME00 | 3089 | 6483 | 1098.1 | 1355.7 | 166.2 | 287.5 | 1.57 |
| Servia | RS00 | 2567 | 15178 | 11035.8 | 19312.2 | 5376.3 | 1315.5 | **20.05** |
| Vzhodna Slovenija | SI03 | 2245 | 6898 | 4375.1 | 7453.4 | 451.9 | 675.2 | 7.01 |
| Zahodna Slovenija | SI04 | 1604 | 4537 | 2455.0 | 3975.5 | 1024.0 | 591.0 | 4.35 |
|  |  |  | % | 28.8 | 59.5 | 7.5 | 4.2 |  |

## Prior Distribution Effort example in the Sava

The prior distribution effort is the second step of the Pressure Reduction Analysis implemented in the ESPRES framework. It show a prior appraisal related to the best way to distribute the pressures reduction considering equal effort to apply and unit of reduction in the different regions and sectors. This step is purely informative and does not affect the pressure reduction analysis. This step offers an initial reference of optimal distribution of the pressure reductions between sectors and administrative regions in a basin where all efforts are set equal, and is informed by one hundred previous optimization executions done with equal efforts. The tool provides three prior references level of pressure reduction, i.e. reducing the median environmental metric at 90%, 75% and 50% of its current status (figure AM 1). These 90%, 75% and 50% are extracted from the 90, 75 and 50 percentile of each Pareto frontier in the one hundred executions.


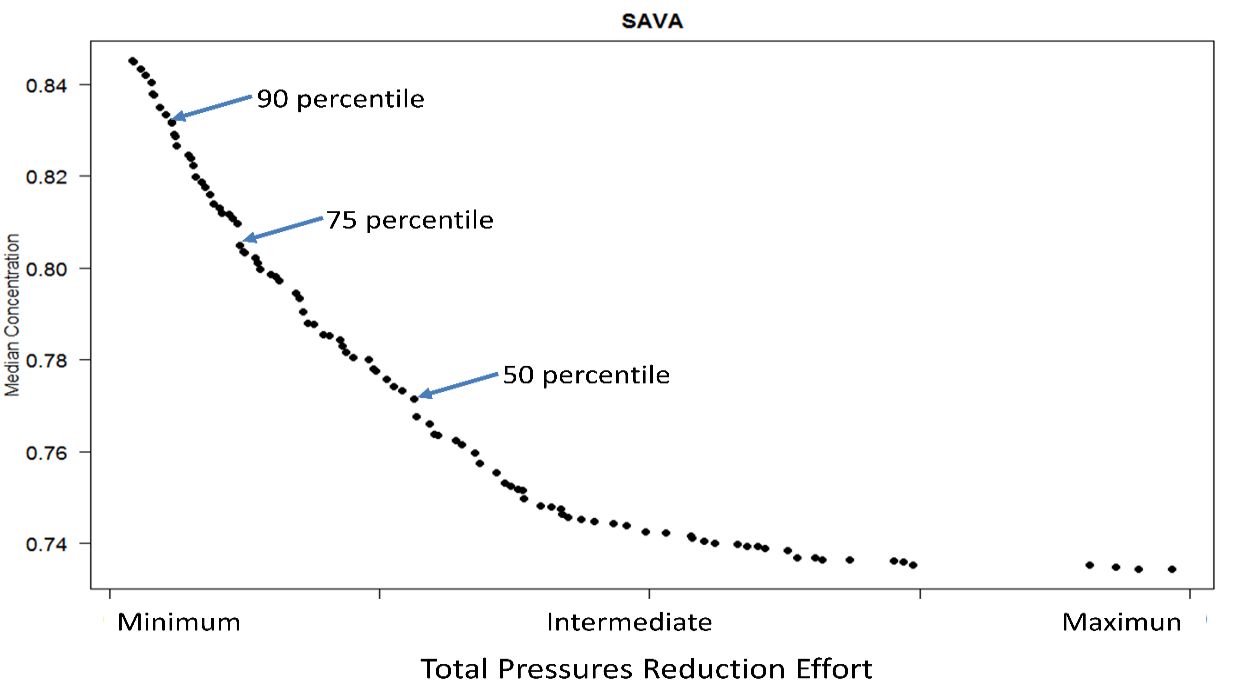


*Figure AM 1: Location in the Pareto frontier of the three prior references level of environmental quality reduction. Metric at 90, 75 and 50th percentile of the Pareto points for the current status.*


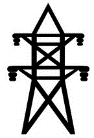


**5**

**1**

**5**

**2**

**4**

***ITH1***

***ITH2***

***ITH3***

**9**

**6**

**8**

**7**

**5**

**3**

**3**

**11**

**8**

**7**

**7**

**7**

**1**

**2**

**6**

***CH05***

Water demand

Reduction (Mm^3^)

Relative Effort

(10^3^€/Mm3)

Effort

(10^3^ €)

**50**

**8**

**10**

**4**

**30**


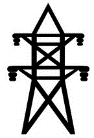


**250**

**8**

**50**

**8**

**120**

***ITH1***

***ITH2***

***ITH3***

**450**

**48**

**80**

**28**

**150**

**150**

**24**

**110**

**32**

**210**

**350**

**56**

**10**

**8**

**180**

***CH05***

**x**

**2072**

10^3^ €


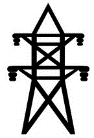

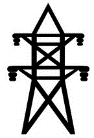


**5**

**1**

**5**

**2**

**4**

***ITH1***

***ITH2***

***ITH3***

**9**

**6**

**8**

**7**

**5**

**3**

**3**

**11**

**8**

**7**

**7**

**7**

**1**

**2**

**6**

***CH05***

Water demand

Reduction (Mm^3^)

Relative Effort

(10^3^€/Mm3)

Effort

(10^3^ €)

**50**

**8**

**10**

**4**

**30**


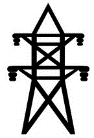


**250**

**8**

**50**

**8**

**120**

***ITH1***

***ITH2***

***ITH3***

**450**

**48**

**80**

**28**

**150**

**150**

**24**

**110**

**32**

**210**

**350**

**56**

**10**

**8**

**180**

***CH05***

**x**

**2072**

10^3^ €


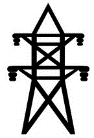


Once the user has selected one of these three reference levels on environmental improvement, he can visualize the distributions of efforts for that situation. These distributions in the application of the efforts are shown by regions, by sectors or by both simultaneously. Boxplot graphs display the distribution of the effort shares between sectors and regions.

For the module of quantity considering the Sava basin, Figure AM2 shows the prior distribution effort plots by regions and figure AM3 shows the prior distribution effort plots by sector. In both figures, the two left plot correspond to the 90th percentile of the Pareto fronts strategies and the two right plots correspond to the 50th percentile of the Pareto fronts strategies. The top two plots show the distribution of efforts by regions in relation to the maximum effort applicable in the region (in this example the maximum applicable in each region and sector was set at 50% of the current emissions). The two plots below show the distribution of regional efforts in relation to the total applicable effort (in the basin).

| 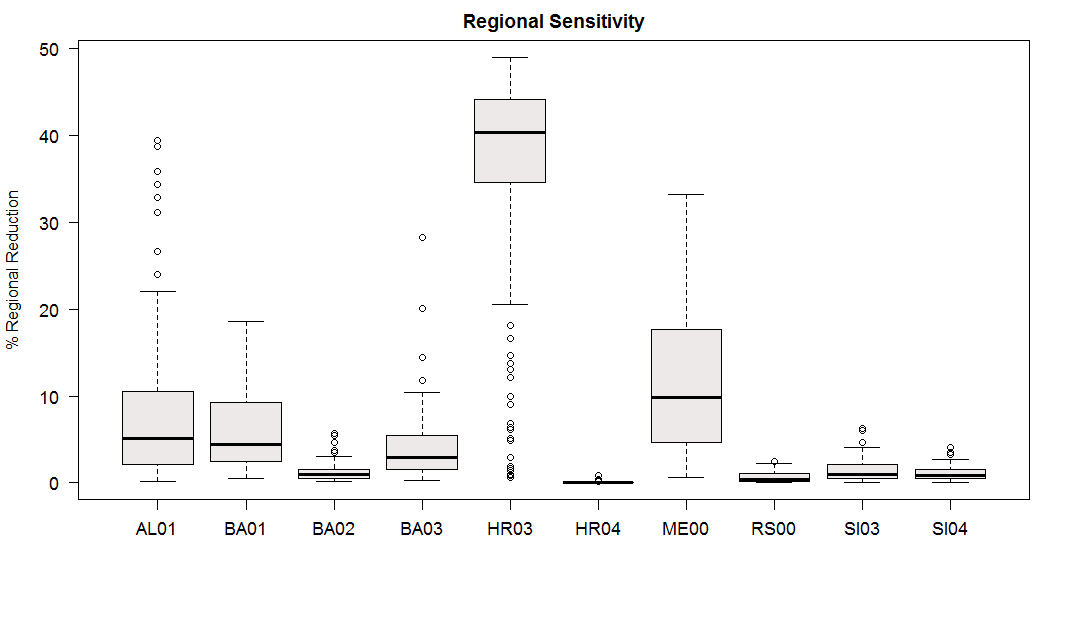 | 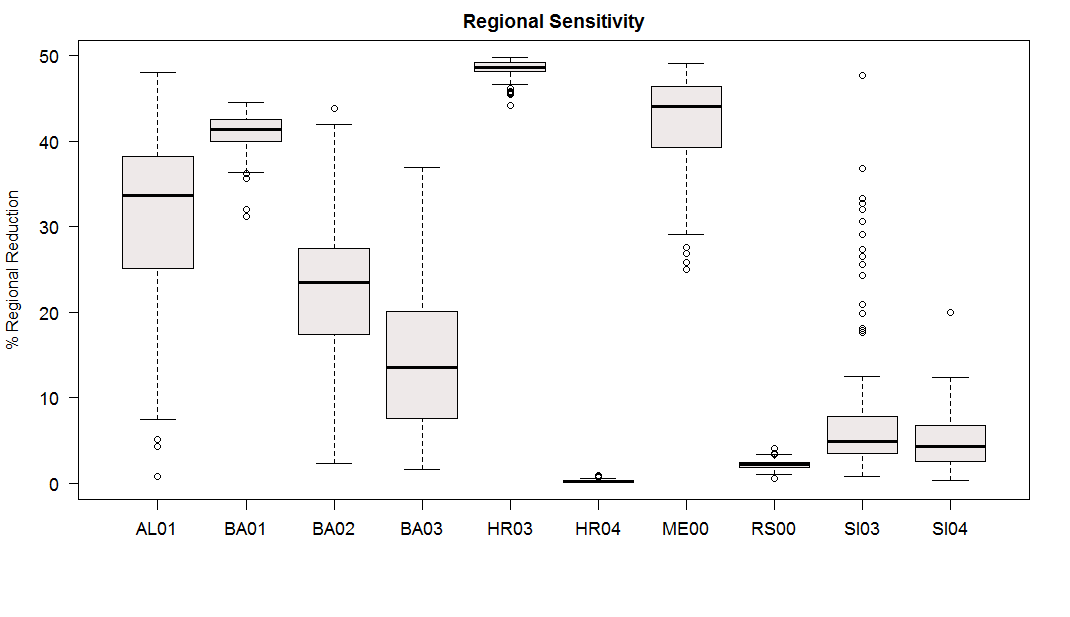 |
| --- | --- |
| 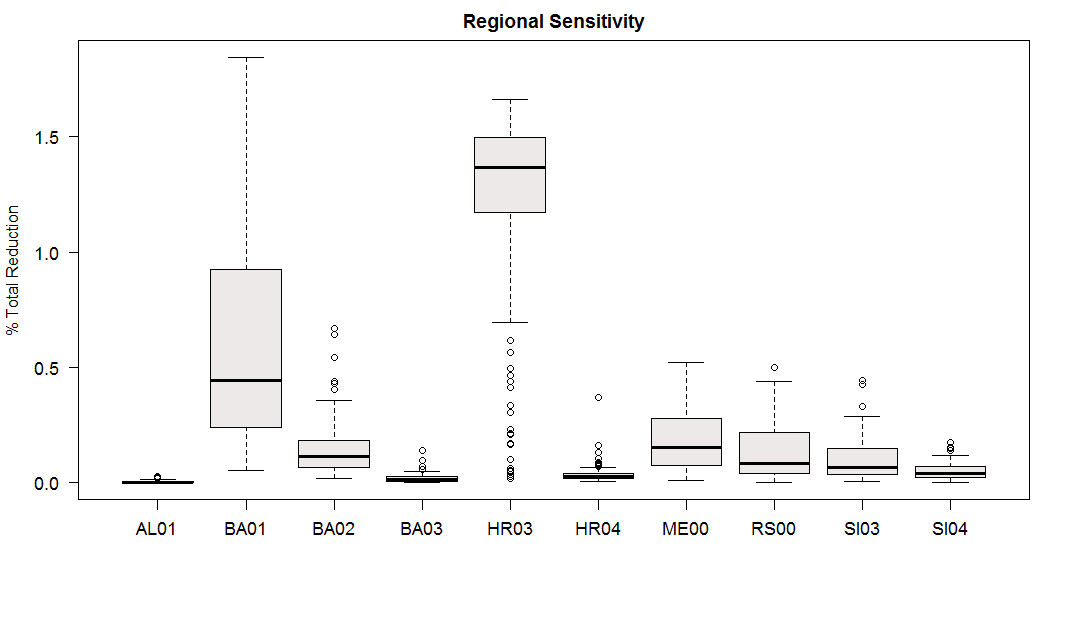 | 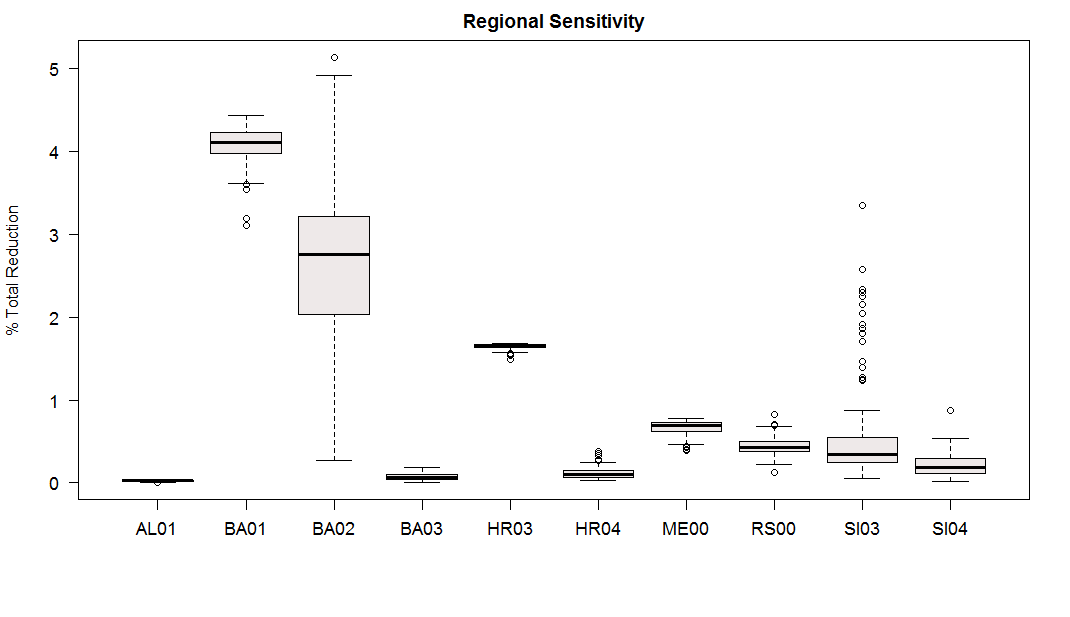 |
|  |  |

*Figure AM 2: Regional Prior Distribution Effort (% pressures reduction) application for the Sava basin. Strategies corresponding to 90th percentile of the Pareto (left) and 50th percentile of the Pareto (right). Top figures show by the relative effort applied in each region according to the maximum of the region. In the bottom figures is show the relative effort applied in each region according to the total maximum effort in the Basin.*


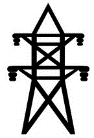


**5**

**1**

**5**

**2**

**4**

***ITH1***

***ITH2***

***ITH3***

**9**

**6**

**8**

**7**

**5**

**3**

**3**

**11**

**8**

**7**

**7**

**7**

**1**

**2**

**6**

***CH05***

Water demand

Reduction (Mm^3^)

Relative Effort

(10^3^€/Mm3)

Effort

(10^3^ €)

**50**

**8**

**10**

**4**

**30**


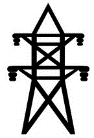


**250**

**8**

**50**

**8**

**120**

***ITH1***

***ITH2***

***ITH3***

**450**

**48**

**80**

**28**

**150**

**150**

**24**

**110**

**32**

**210**

**350**

**56**

**10**

**8**

**180**

***CH05***

**x**

**2072**

10^3^ €


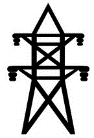

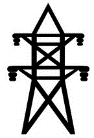


**5**

**1**

**5**

**2**

**4**

***ITH1***

***ITH2***

***ITH3***

**9**

**6**

**8**

**7**

**5**

**3**

**3**

**11**

**8**

**7**

**7**

**7**

**1**

**2**

**6**

***CH05***

Water demand

Reduction (Mm^3^)

Relative Effort

(10^3^€/Mm3)

Effort

(10^3^ €)

**50**

**8**

**10**

**4**

**30**


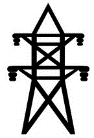


**250**

**8**

**50**

**8**

**120**

***ITH1***

***ITH2***

***ITH3***

**450**

**48**

**80**

**28**

**150**

**150**

**24**

**110**

**32**

**210**

**350**

**56**

**10**

**8**

**180**

***CH05***

**x**

**2072**

10^3^ €


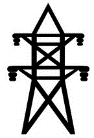


The HR03 (Jadranska), the ME00 (Montenegro) and the BA01 (Bosnei) are the regions where the optimal strategies (of the one hundres previous executions) propose to apply practically the maximum limit of pressure reduction (top right plot in figure AM 2, corresponding to 50th percentile strategies), probably because they are upstream. In the overall of the basin for the 50th percentile strategies (figure AM 2 bottom right) only the BA01 effort counts for approximately a 4% of the total reduction, greater reduction than that achieved for all regions of Sava (except BA02).

Examining then the results by sectors, considering what is appreciated in table AM 1 (59% is mineral, 29% is manure, PS and SD add up to 12%) it is appreciated as sectorial it seems that first it proposes to apply most relative effort in SD (figure AM 3 top right), but under the total Sava perspective (figure AM 3 bottom right) the mineral and the manure are the type of nutrient were efficient strategies propose to effort that achieves reduction around the 5%.

| 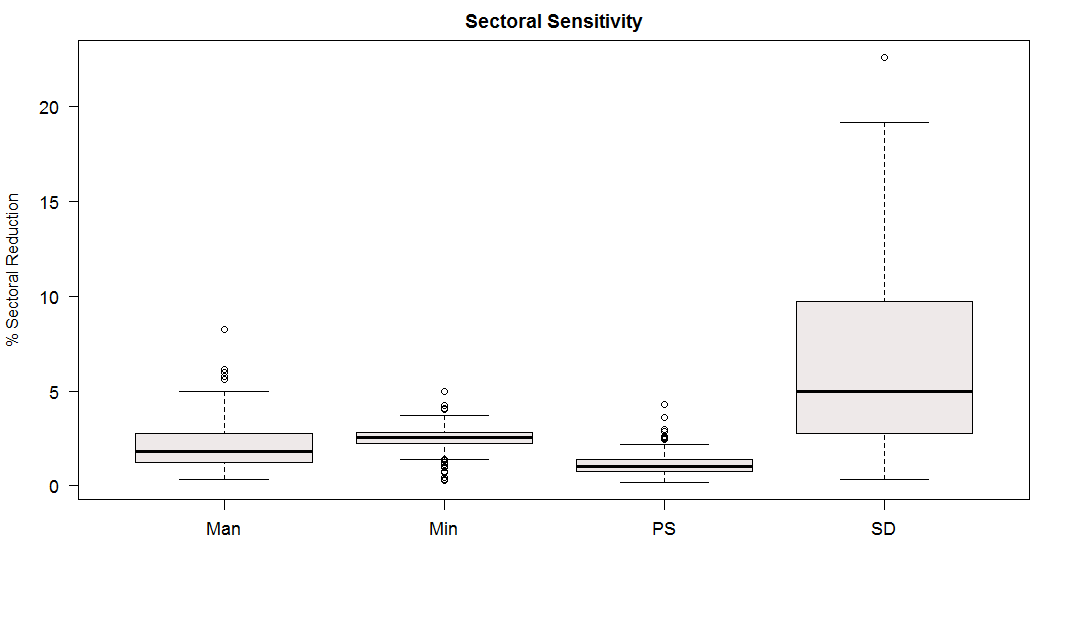 | 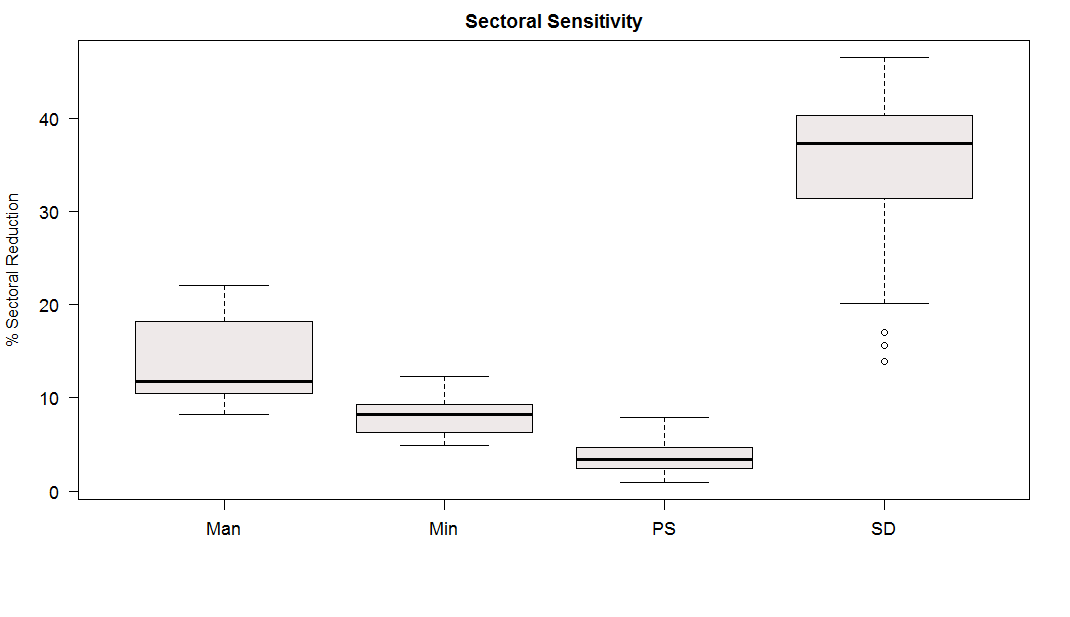 |
| --- | --- |
| 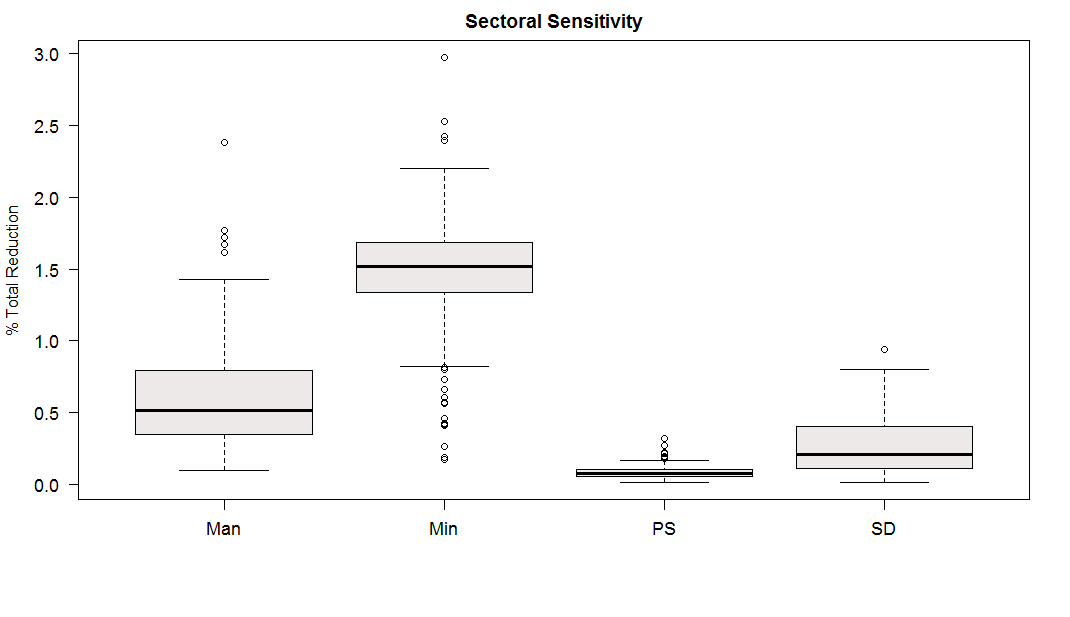 | 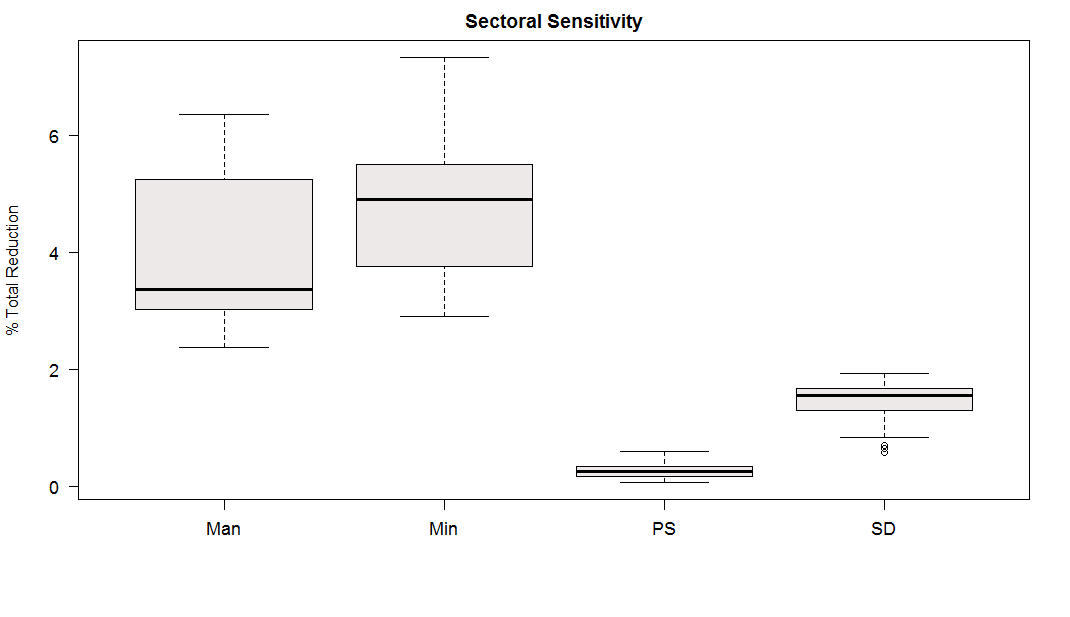 |

*Figure AM 3: Sectoral Prior Distribution Effort (% pressures reduction) application for the Sava basin. Strategies corresponding to 90th percentile of the Pareto (left) and 50th percentile of the Pareto (right). Top figures show by the relative effort applied in each region according to the maximum of the region. In the bottom figures is show the relative effort applied in each region according to the total maximum effort in the Basin.*


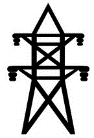


**5**

**1**

**5**

**2**

**4**

***ITH1***

***ITH2***

***ITH3***

**9**

**6**

**8**

**7**

**5**

**3**

**3**

**11**

**8**

**7**

**7**

**7**

**1**

**2**

**6**

***CH05***

Water demand

Reduction (Mm^3^)

Relative Effort

(10^3^€/Mm3)

Effort

(10^3^ €)

**50**

**8**

**10**

**4**

**30**


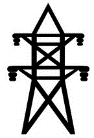


**250**

**8**

**50**

**8**

**120**

***ITH1***

***ITH2***

***ITH3***

**450**

**48**

**80**

**28**

**150**

**150**

**24**

**110**

**32**

**210**

**350**

**56**

**10**

**8**

**180**

***CH05***

**x**

**2072**

10^3^ €


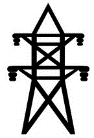

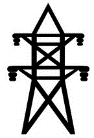


**5**

**1**

**5**

**2**

**4**

***ITH1***

***ITH2***

***ITH3***

**9**

**6**

**8**

**7**

**5**

**3**

**3**

**11**

**8**

**7**

**7**

**7**

**1**

**2**

**6**

***CH05***

Water demand

Reduction (Mm^3^)

Relative Effort

(10^3^€/Mm3)

Effort

(10^3^ €)

**50**

**8**

**10**

**4**

**30**


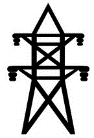


**250**

**8**

**50**

**8**

**120**

***ITH1***

***ITH2***

***ITH3***

**450**

**48**

**80**

**28**

**150**

**150**

**24**

**110**

**32**

**210**

**350**

**56**

**10**

**8**

**180**

***CH05***

**x**

**2072**

10^3^ €


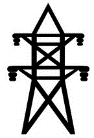


Finally, the prior distribution effort tool show it jointly by regions and sectors (regional sector). (Fig. AM 4). Where can be observed how the effort applied in reduce manure and mineral in BA01 region accounts approximately for the 2% and 1.5% of the global reduction respectively when consider the results of the 50 th percentile executions. While up to the 90th percentile strategies focus to reduce Mineral in HR03 (figure AM4 left).

| 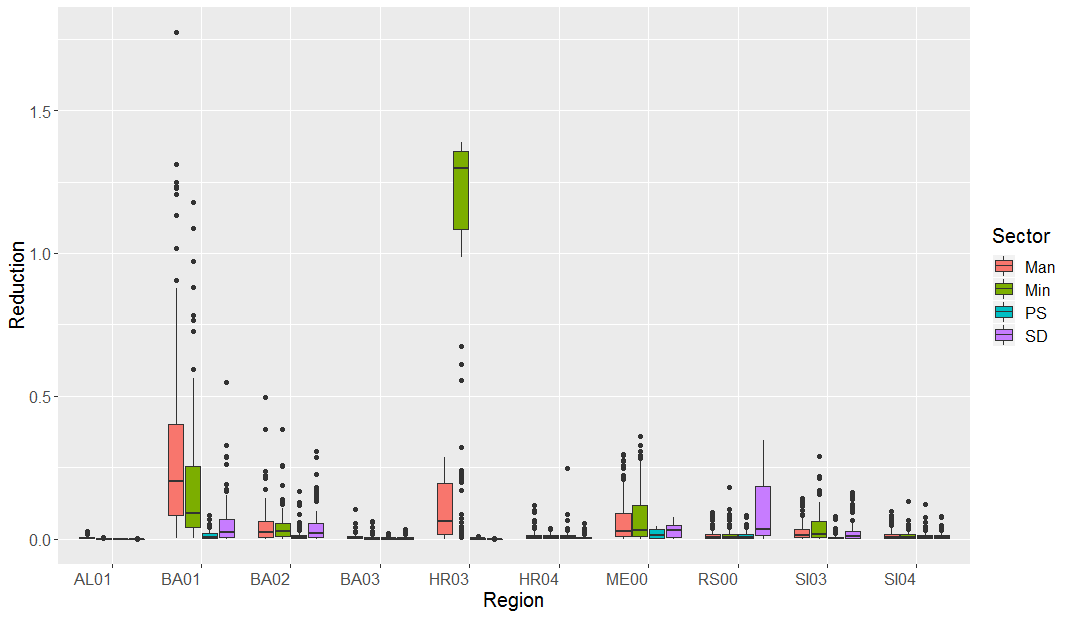 | 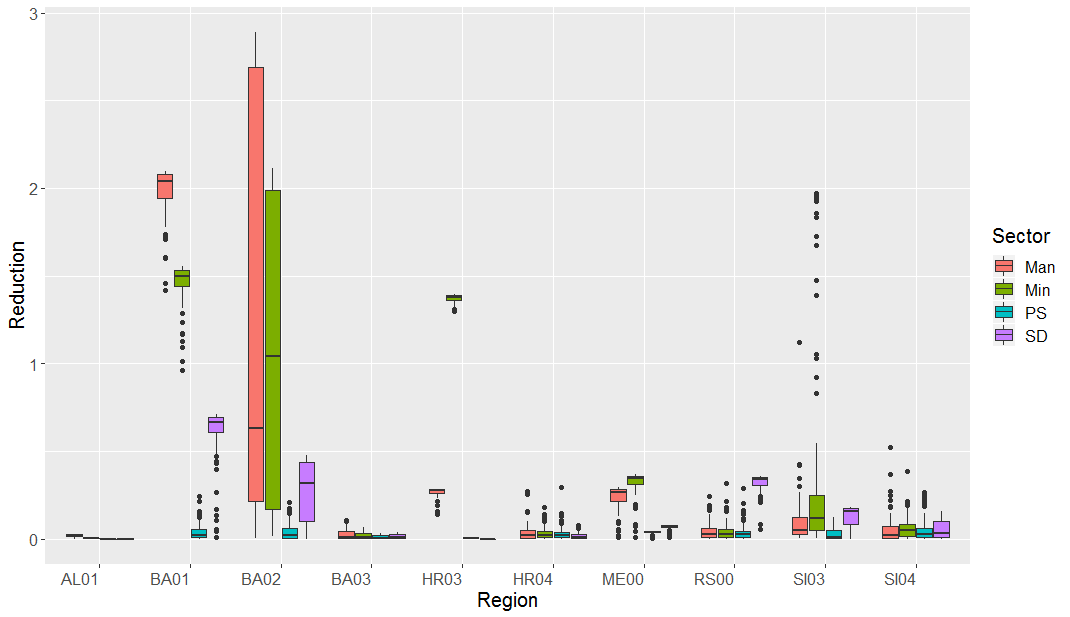 |
| --- | --- |

*Figure AM 4: Sectoral & Regional Prior Distribution Effort (% pressures reduction) application for the Sava basin. Strategies corresponding to 90th percentile of the Pareto (left) and 50th percentile of the Pareto (right).*


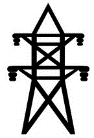


**5**

**1**

**5**

**2**

**4**

***ITH1***

***ITH2***

***ITH3***

**9**

**6**

**8**

**7**

**5**

**3**

**3**

**11**

**8**

**7**

**7**

**7**

**1**

**2**

**6**

***CH05***

Water demand

Reduction (Mm^3^)

Relative Effort

(10^3^€/Mm3)

Effort

(10^3^ €)

**50**

**8**

**10**

**4**

**30**


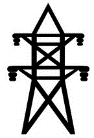


**250**

**8**

**50**

**8**

**120**

***ITH1***

***ITH2***

***ITH3***

**450**

**48**

**80**

**28**

**150**

**150**

**24**

**110**

**32**

**210**

**350**

**56**

**10**

**8**

**180**

***CH05***

**x**

**2072**

10^3^ €


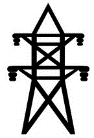

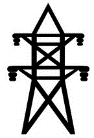


**5**

**1**

**5**

**2**

**4**

***ITH1***

***ITH2***

***ITH3***

**9**

**6**

**8**

**7**

**5**

**3**

**3**

**11**

**8**

**7**

**7**

**7**

**1**

**2**

**6**

***CH05***

Water demand

Reduction (Mm^3^)

Relative Effort

(10^3^€/Mm3)

Effort

(10^3^ €)

**50**

**8**

**10**

**4**

**30**


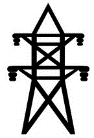


**250**

**8**

**50**

**8**

**120**

***ITH1***

***ITH2***

***ITH3***

**450**

**48**

**80**

**28**

**150**

**150**

**24**

**110**

**32**

**210**

**350**

**56**

**10**

**8**

**180**

***CH05***

**x**

**2072**

10^3^ €


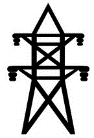


## Environmental models details

The long term mean annual water availability to compute the WEI is estimated with a Budyko approach (Budyko 1974). Budyko model provides long-term water availability in a natural basin based on its climate and land cover. For European scale application, we refer to the variant proposed by Zhang et al., 2001:

| $ET=P-\frac{P+wE_{0}}{1+w\frac{E_{0}}{P}+\frac{P}{E_{0}}}$ |  |
| --- | --- |

Where ET is long-term mean annual evapotranspiration, P is long-term mean annual precipitation, E_0_ is potential evapotranspiration and w is a parameter termed the plant-available water coefficient (Zhang et al., 2001) representing the relative difference in the way plants use soil water for transpiration. In this analysis, we assume the average of E_0_ and P over the period 1990-2010 to represent current conditions. The term E_0_/P$\frac{E_{0}}{P}$, sometimes called the “aridity index”, depends only on the climate of a region, while w may be related to vegetation and land cover.

The total water demand in the spatial unit is estimated for five sectors. Domestic, industrial and energy demands are derived according to Vandecasteele et al. (2014). Livestock breeding water demand is estimated by Mubareka et al. (2013). The irrigation demand is estimated as discussed in Pistocchi et al. (2018). The year of reference for water demand is 2006, and we assume that no significant change in spatial patterns and intensity of water demand has occurred since.

TN is modelled with GREEN (Grizzetti et al., 2012), which is a European conceptual model that considers nitrogen emissions from diffuse sources (fertilization in agricultural land, soil and plant fixation, and background atmospheric deposition) and point sources (including domestic and industry emissions). Model spatial units are sub-catchments of about 7 km^2^. The model accounts for land retention of diffuse sources, i.e. retention of nutrients in the sub-catchment before being discharged to the stream network, and stream retention, which occurs in the stream network for all nutrients entering the reaches. Land retention is modelled through an exponential function that is inversely proportional to annual rainfall; stream retention is modelled through an exponential function of reach length. The two exponential retention factors are calibration parameters. The model provides annual Total Nitrogen loads, which are transformed into mean annual concentration on the basis of the mean annual discharge assessed with the European hydrological model Lisflood (de Roo et al., 2012). Differences in model annual results reflect differences in precipitation, which affects land retention, and in the Lisflood annual discharge. For application in ESPRES, GREEN inputs and outputs were set for the period 2005-2012. Point source emissions were set accordingly to conditions for the 2010s (Vigiak et al., 2018) and were kept constant in the years. Agricultural land and fertilization levels were set according to the nitrogen fertilizer applications estimated by the model CAPRI (https://www.capri-model.org/dokuwiki/doku.php), spatialized using the land cover information of CORINE Land Cover Map 2012 (https://land.copernicus.eu/pan-european/corine-land-cover). Annual data of atmospheric deposition were taken from the EMEP [2001].


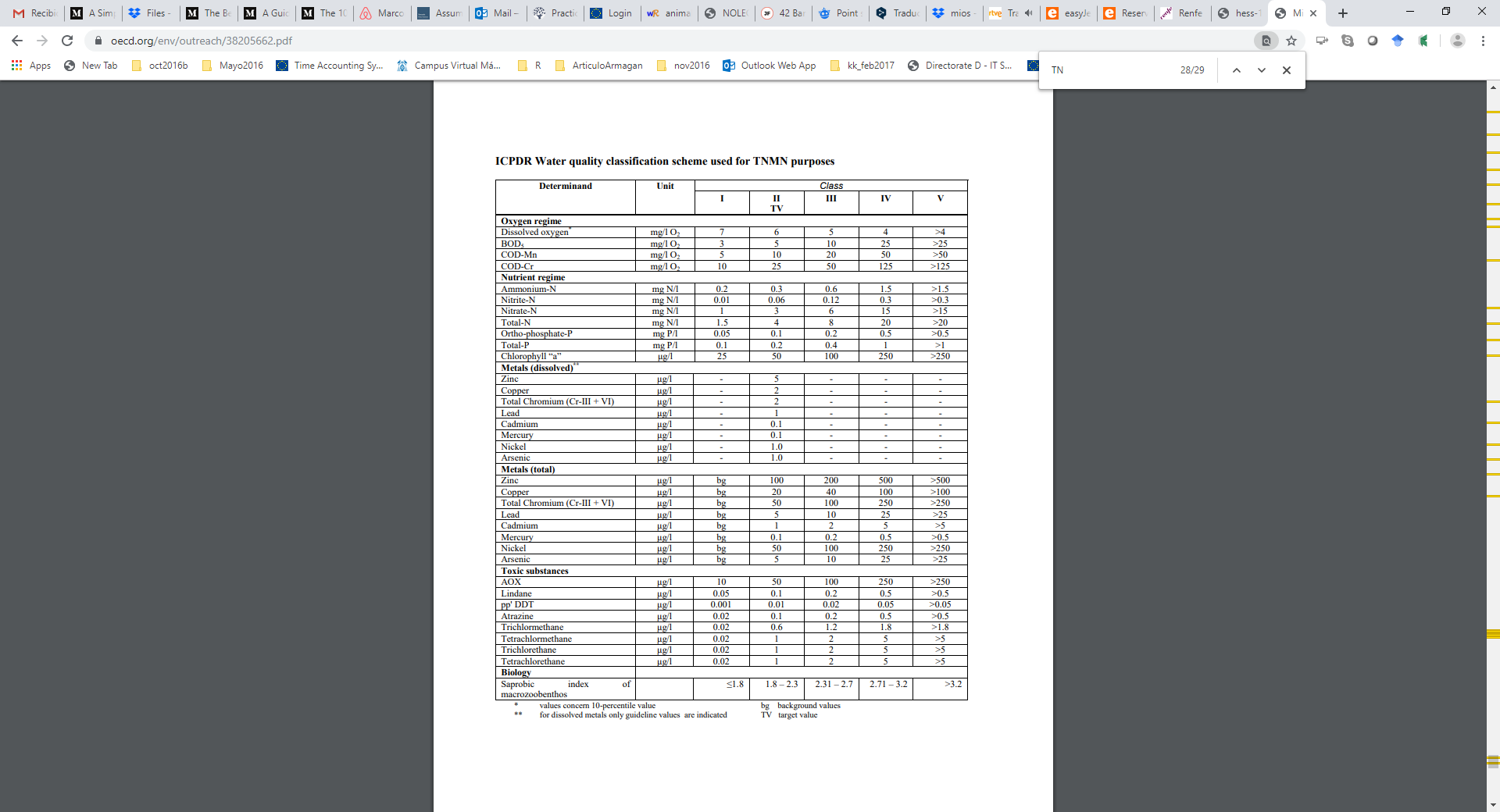

Supplement: Supplementary file 1 — Additional Data and Environmental Model Details [file mmc1.docx]
